# OntoPharma: ontology based clinical decision support system to reduce medication prescribing errors

**DOI:** 10.1186/s12911-022-01979-3

**Published:** 2022-09-10

**Authors:** Elena Calvo-Cidoncha, Concepción Camacho-Hernando, Faust Feu, Xavier Pastor-Duran, Carles Codina-Jané, Raimundo Lozano-Rubí

**Affiliations:** 1 Pharmacy Service, Division of Medicines, Hospital Clínic of Barcelona, 170 Villarroel Street, 08036 Barcelona, Spain; 2 Division of Medicines, Hospital Clínic of Barcelona, 170 Villarroel Street, 08036 Barcelona, Spain; 3 Management Team, Hospital Clínic of Barcelona, 170 Villarroel Street, 08036 Barcelona, Spain; 4 Unit of Medical Informatics, Hospital Clínic of Barcelona, 170 Villarroel Street, 08036 Barcelona, Spain

**Keywords:** “Biological ontologies” [Mesh], “Decision support systems clinical” [Mesh], “Medication errors” [Mesh], “Drug prescriptions” [Mesh]

## Abstract

**Background:**

Clinical decision support systems (CDSS) have been shown to reduce medication errors. However, they are underused because of different challenges. One approach to improve CDSS is to use ontologies instead of relational databases. The primary aim was to design and develop OntoPharma, an ontology based CDSS to reduce medication prescribing errors. Secondary aim was to implement OntoPharma in a hospital setting.

**Methods:**

A four-step process was proposed. (1) Defining the ontology domain. The ontology scope was the medication domain. An advisory board selected four use cases: maximum dosage alert, drug-drug interaction checker, renal failure adjustment, and drug allergy checker. (2) Implementing the ontology in a formal representation. The implementation was conducted by Medical Informatics specialists and Clinical Pharmacists using Protégé-OWL. (3) Developing an ontology-driven alert module. Computerised Physician Order Entry (CPOE) integration was performed through a REST API. SPARQL was used to query ontologies. (4) Implementing OntoPharma in a hospital setting. Alerts generated between July 2020/ November 2021 were analysed.

**Results:**

The three ontologies developed included 34,938 classes, 16,672 individuals and 82 properties. The domains addressed by ontologies were identification data of medicinal products, appropriateness drug data, and local concepts from CPOE. When a medication prescribing error is identified an alert is shown. OntoPharma generated 823 alerts in 1046 patients. 401 (48.7%) of them were accepted.

**Conclusions:**

OntoPharma is an ontology based CDSS implemented in clinical practice which generates alerts when a prescribing medication error is identified. To gain user acceptance OntoPharma has been designed and developed by a multidisciplinary team. Compared to CDSS based on relational databases, OntoPharma represents medication knowledge in a more intuitive, extensible and maintainable manner.

**Supplementary Information:**

The online version contains supplementary material available at 10.1186/s12911-022-01979-3.

## Background

Medication errors are a serious public health problem and a leading cause of high morbidity and mortality [1–6]. Medication management is a complex multistep process. Errors can occur at any step, from prescribing to administering the medication. However, studies have shown that drug prescription errors are the most frequent [7, 8].

Clinical decision-making at the time of prescription depends on the ability of the clinician to collect and evaluate all patient data to make the most appropriate decision that improves their health outcomes [9]. The segmentation of health services and the complexity, volume and dynamics of clinical information are factors that increase the likelihood of a medication error.

Approaches involving information systems, such as computerised physician order entry (CPOE) [10] combined with clinical decision support systems (CDSS) [11] have been shown to reduce drug prescription errors.

CDSS link patient data with a knowledge base to generate information that help clinician make decisions [12]. Relational databases are in most cases the system of choice when it comes to designing a CDSS. The potential of CDSS to reduce medication errors is clear. However, they are underused. There is growing literature about why clinicians fail to utilize CDSS suggestions [13]. Lack of interoperability or alert fatigue explain high alert override rates [14–17]. Another challenge is the maintenance of the knowledge base up to date with the literature-based and practice-based evidence [18].

In order to overcome the challenges described above, one approach to improve CDSS is to use ontologies instead of relational databases [19].

An ontology is an explicit conceptualization of the entities of a domain. It includes machine-interpretable definitions of concepts in the domain and relations among them [20, 21]. Since ontologies define the terms used to describe and represent an area of knowledge, they are used in many applications to facilitate data annotation, information retrieval or aid in education [22, 23]. Ontologies have the potential to support the development of CDSS in a manner that enhances reusability of data and knowledge. There are already existing ontology based CDSS representing a wide range of medical domains [24]. However, only a few are addressed to medication management and usually, they are restricted to a specific disease or specialty.

The primary aim was to design and develop OntoPharma, an ontology based CDSS to reduce medication prescribing errors. Secondary aim was to implement OntoPharma in a hospital setting.

## Methods

The study was conducted between 2016 and 2021 at a 710-bed tertiary hospital in Spain equipped with CPOE and an Electronic Health Record (EHR) system provided by SAP®. A four-step development process was designed: defining the ontology domain and scope; implementing the ontology in a formal representation; developing an ontology-driven alert module and implementing OntoPharma in a hospital setting.

### Defining the ontology domain and scope

The ontology scope focused on the medication domain. Given the range and complexity of the domain, a multidisciplinary advisory board selected four use cases: maximum dosage alert, drug-drug interaction checker, renal failure adjustment, and drug allergy checker.

We used three different sources of information:

#### Nomenclator for prescription

The dataset was provided by the Spanish Agency of Medicines and Medical Devices (AEMPS) [25]. It contains identification and technical data of all medicinal products that have been authorised and marketed, financed and unfunded.

#### Agency for Health Quality and Assessment of Catalonia (AQuAS) [26]

AQuAS offers different datasets with information to improve medication safety. These datasets are collected and reviewed periodically by experts in the field of medication safety.

Maximum daily dose dataset contained 1013 entries. The dataset included maximum daily doses for high-risk medications. Each entry consisted of the following fields: code type (Anatomical Therapeutic Chemical code (ATC), National Drug Code, SNOMED CT code); code description; route of administration; maximum daily dose and unit; indication (just if dosage differs for different indications); age range; gravity; alert description; recommendation and bibliography.

Drug-drug interactions dataset contained 3229 entries. Each entry referred to a pair of drug-drug interactions and consisted of the following fields: code type (ATC, National Drug Code, SNOMED CT code); code description; route of administration; gravity; alert description; recommendation and bibliography.

#### ABX dosage

The dataset was provided by local experts in medication renal failure adjustment and contained 179 entries of antimicrobial agents. Each entry consisted of the following fields: drug description (non-standardized); route of administration; dosage by clearing interval; dosage for patients on dialysis and bibliography.

### Implementing the ontology in a formal representation

Nomenclator for Prescription contains structured data in xml format. Contents from other resources are semi-structured data. Prior to modelling drug-related knowledge through ontologies, we processed all the information in a relational database to clean the data, detect redundancies and detect relationships between different concepts.

The design, development and maintenance of the ontologies have been driven by Medical Informatics specialists and Clinical Pharmacists. The information was represented in the Web Ontology Language (OWL) [27]. For encoding the OWL ontologies, we used the Protégé 3.5 editor tool [28].

The concepts of the medication domain were organized hierarchically following a top-down approach. This process starts with the definition of the most general concepts in the domain and subsequent specialization of the concepts. The class hierarchy development, defining properties and slots of concepts were carried out at the same time. Finally, we defined individual instances of the classes represented.

In any case, ontology development is an iterative process based on the review of the state of the art which continues through the entire lifecycle of the ontology.

### Ontology-driven alert module development

The integration between the CPOE system and the ontologies was performed through a REST API. A REST API call is published (in JSON format) each time a clinician adds a new medication in the CPOE, modify an existing one or request on demand CDSS information.

The request contains patient-specific clinical data: demographic data, prescription data, laboratory parameters and history of drug allergy. Our EHR does not use standardized terminology. In order to ensure interoperability, local concepts were manually mapped with existing concepts in the ontologies.

SPARQL was used to query ontologies [29]. SPARQL lets pull values from structured data by utilizing a set of semantic relationships. SPARQL queries were used to check the appropriateness of the prescription using the assertional knowledge represented in the ontologies and generate a set of alerts if needed. We have used Apache Jena Fuseki as SPARQL Server due to its ease of installation and configuration [30]. This application allows to make consultations and remote updating/modifications using SPARQL 1.1. protocol. After applying the queries, a returning REST API with the results is published.

Alerts are shown in the CPOE only in case of overdosing, drug-drug interactions, dose adjustment for renal failure required or allergies. The final user interface was designed by the advisory board to ensure usability and the minimum interference with the clinician workflow. A formal testing was performed to demonstrate that the ontology-driven alert module met functional requirements. Manual testing was also performed. Clinical Pharmacists interacted with all the prescribing alerts represented in OntoPharma in a control environment (SAP-QAS®).

A relational database management system (MariaDB) is used for auditing. Each input and output data are recorded in order to allow traceability.

### OntoPharma implementation in a hospital setting

In July 2020, OntoPharma was implemented at one ward of Internal Medicine Unit with capacity for twenty admissions. Informatics staff and Clinical Pharmacists were responsible for the diffusion and for providing support.

A retrospective analysis of the alerts generated by OntoPharma was performed. We included patients admitted to Internal Medicine ward from July 2020 to November 2021. The following patient data were collected: gender, age, duration of hospital stay and number of medications during hospital stay. We further examined the alerts including the number, type of alert, clinical relevance and the acceptance rate.

Quantitative variables are expressed as mean and standard deviation or as median and percentile P25 and P75 in case of a skewed distribution. Qualitative variables are expressed as percentages. Data analysis was carried out using SPSS 20.0.

## Results

### Knowledge representation using ontologies

For modelling drug-related knowledge we have developed a total of three ontologies. Each ontology has been divided into two parts. The first part provides concepts/classes (also known as T-Box) and the second provides the instances of these concepts (also known as A-Box).

Table 1 shows the details about the name and the domain of ontologies.

**Table 1:** Description of the ontologies used in OntoPharma

Ontology name	Ontology domain
Drugs	Identification and technical data of medicinal products
DSS	Appropriateness drug data
Local_Pharmacy	Local concepts from electronic health record and computerised physician order entry

*DSS* decision support system

**Fig. 1:**
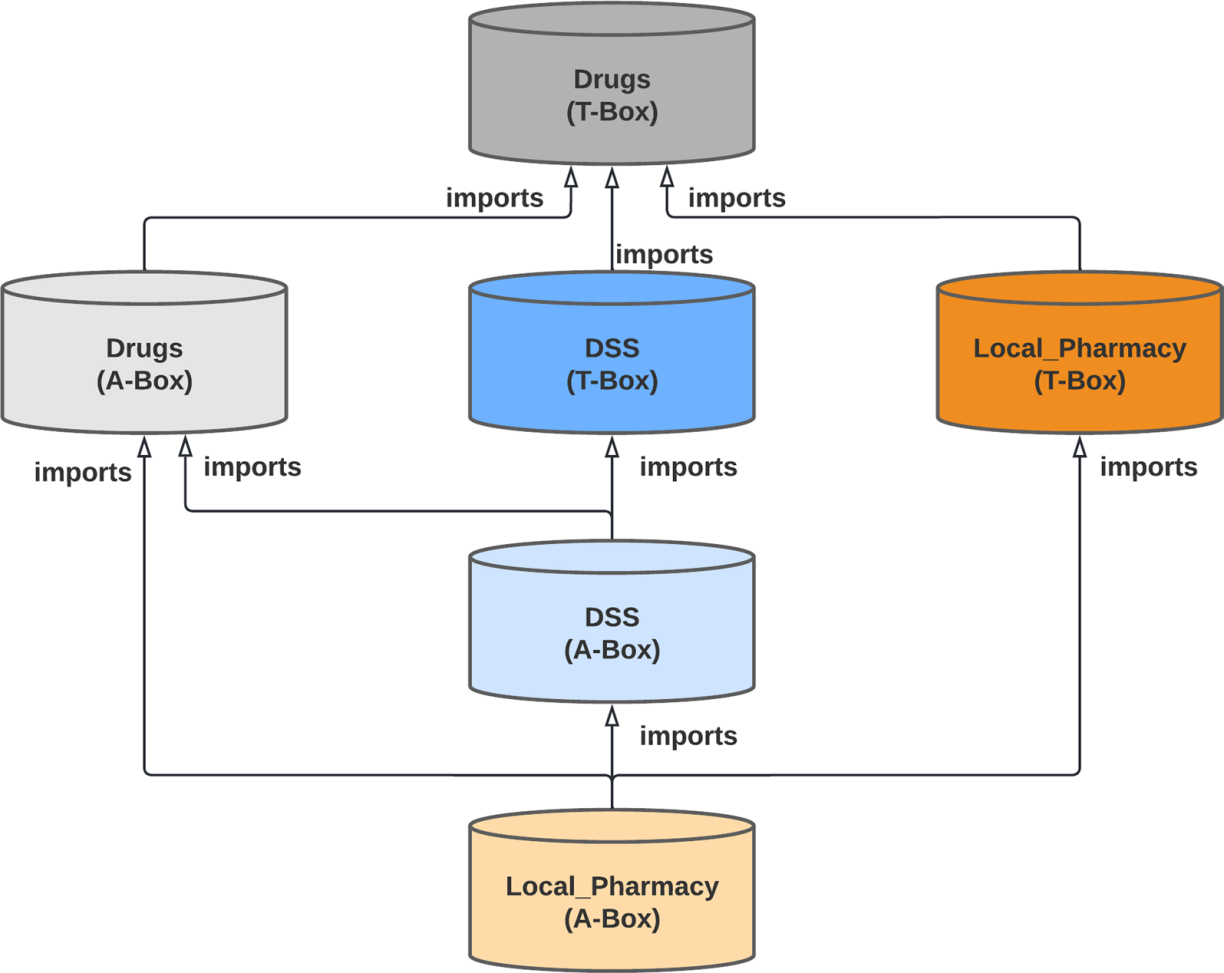
Import schema of the ontologies used in OntoPharma

The three ontologies are interconnected. Import schema of ontologies is shown in Fig. 1. This means concepts described in the ontologies depend on other concepts previously defined. For example, Dose appropriateness concept (DSS ontology) is linked to Unit concept (Drug ontology) through object properties.

In total we have proposed 34,938 classes, 16,672 individuals and 82 properties. See Additional file 1 for full list of the medication knowledge concepts and their definitions. Additional file 2 displays a list of properties and their facets. It should be noted that classes are linked between them through object properties and that slots are attached at the most general class that can have that property. For instance, ingredient is attached at the class Drug appropriateness, so ingredient is inherited to all subclasses of Drug appropriateness, including Maximum dose adult class and Renal adjustment class.

#### Drugs ontology

**Fig. 2:**
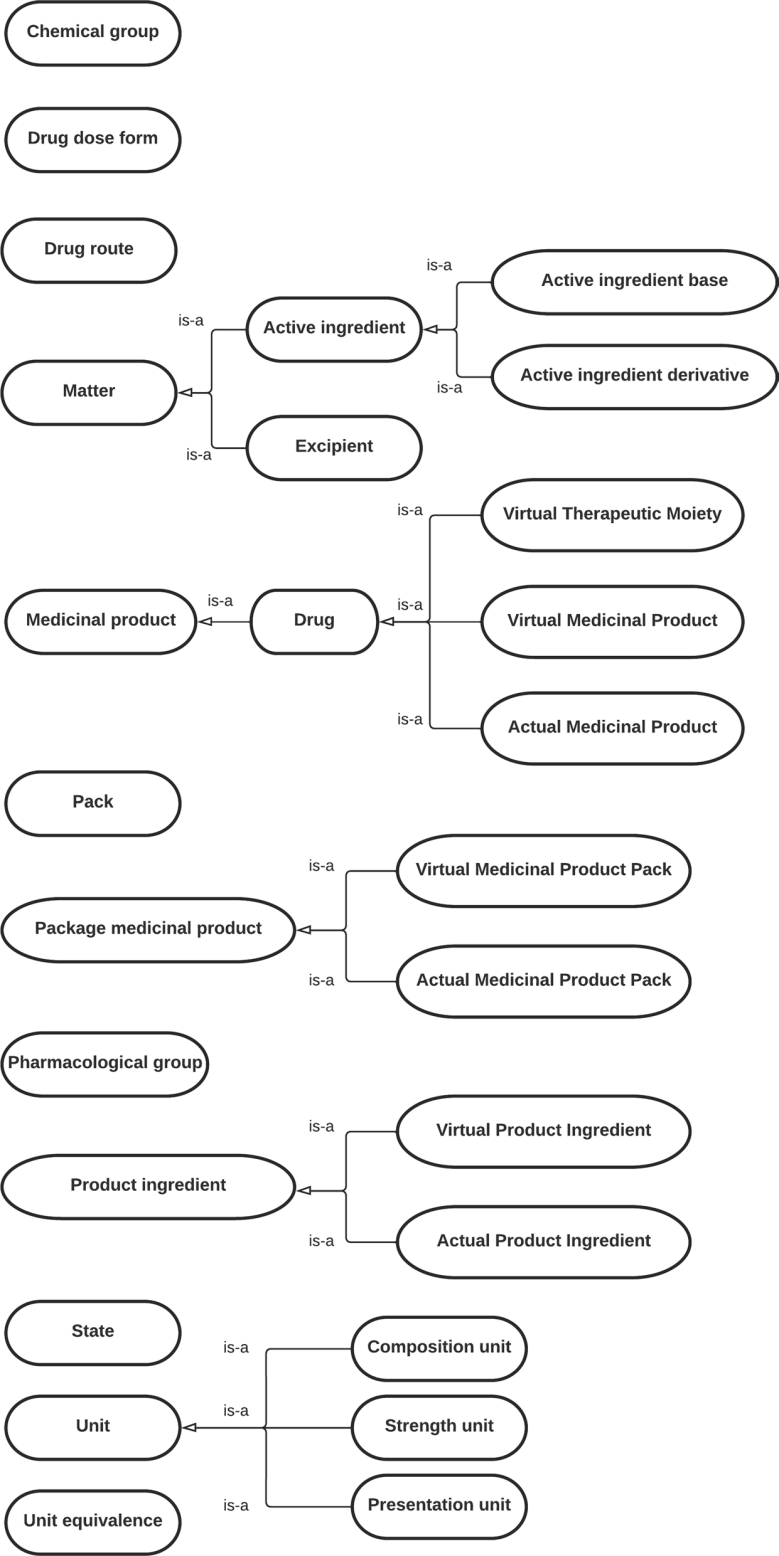
Diagram showing relationships between classes in drugs ontology

Drugs ontology was designed to represent the identification and technical data of medicinal products. Figure 2 provides a diagram showing the class interactions.

The most notable classes are the following: Medicinal Product; Package Medicinal Product and Product ingredient.

Medicinal products are substances which has a physiological effect when are administered. Depending on the detail there are three medicinal products: Virtual Therapeutic Moiety (VTM); Virtual Medicinal Product (VMP) and Actual Medicinal Product (AMP).

A VTM is an abstract representation of an active medicinal ingredient or substance devoid of strength and form (omeprazole). A VMP is a representation of a VTM associated with strength information and a route of administration (omeprazole 20 mg capsule). An AMP is a medicinal product that has been made available by a supplier. It is the medicinal product that is taken by a patient (omeprazole Pfizer 20 mg capsule).

Package medicinal product includes two types of products: Virtual Medicinal Product Pack (VMPP) and Actual Medicinal Product Pack (AMPP).

A VMPP is an abstract concept representing one or more quantitatively equivalent AMPP (omeprazole 20 mg 28 capsules). An AMPP is the commercially produced packaged product which is supplied for direct patient use (omeprazole Pfizer 20 mg 28 capsules). The concept contains information on the pack size, the inner packaging, price and reimbursement information, and other administrative information.

Information of VTM, VMP and VMPP was provided using SNOMED CT terminology.

Product ingredient is any substance used in a pharmaceutical product, intended to furnish pharmacological activity or to otherwise have direct effect in the diagnosis, cure, mitigation, treatment or prevention of disease. Ingredients used in VMP are virtual product ingredient (VPI). Ingredients used in AMP are actual product ingredient (API). API and VPI relate to the same ingredient (omeprazole sodium) but are identified by different code numbers.

Other concepts represented were pharmacological group (ATC_A02BC01), drug dose form (capsule), drug route (oral), pack (carton), state (authorized and marketed), chemical group (proton pump inhibitors), excipient (sucrose) and unit (mg).

#### DSS ontology

**Fig. 3:**
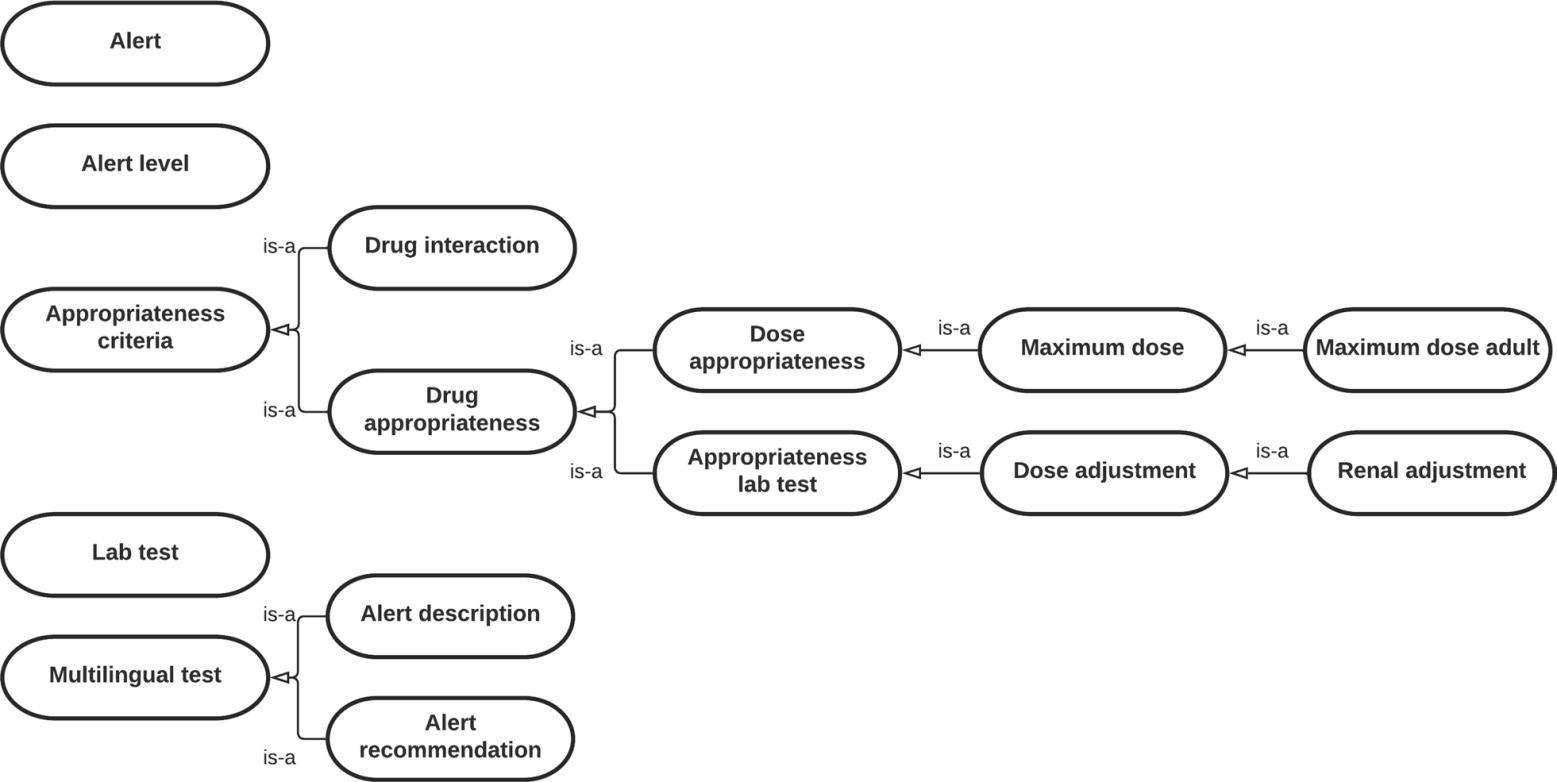
Diagram showing relationships between classes in DSS ontology

DSS ontology provides appropriateness drug data. Figure 3 provides a diagram showing the class interactions.

The most notable classes are the following: Appropriateness criteria and Alert.

Appropriateness criteria include criteria to ensure safe use of medicines in three use cases: maximum dosage alert, drug-drug interaction checker and renal failure adjustment.

Appropriateness criteria class has two subclasses, drug interaction and drug appropriateness.

Drug interaction class contains 2242 individuals defined at different ATC levels. Each individual only contains the ATC for both drugs (ATC N06AF and ATC R05FB02) and the alert.

Drug appropriateness class include criteria based on medication dose (Dose appropriateness) and criteria based on lab test (Appropriateness lab test).

Maximum dose adult class, subclass of dose appropriateness, contains 562 individuals. Each individual contains the following knowledge: ingredient (atorvastatin), route of administration (oral), maximum dose (80), unit (mg), base unit (every 24 h), age range (18–99), and alert.

Renal adjustment class, subclass of appropriateness lab test, contains 268 individuals. Each individual contains the following knowledge: ingredient (dalvabancin), route of administration (parenteral), laboratory test (glomerular filtration rate (GFR)), laboratory test unit (ml/min/1.73 m2), low GFR value (31), high GFR value (130), adjusted loading dose (1000), adjusted loading unit (mg), adjusted loading base unit (every 24 h), minimum adjusted dose (500), maximum adjusted dose (500), adjusted dose unit (mg), adjusted base unit (every 24 h) and alert.

Regarding the fourth use case, drug allergy checker, the allergens were defined as subclasses of the matter class (Drug ontology). We represented two levels, chemical group (proton pump inhibitors) and ingredients (omeprazole). Thus, patients with an allergic reaction to a chemical group are supposed to be allergic to all ingredients included in that group.

Alert class include the displayed information when appropriateness criteria are not met. It contains 4533 individuals. Each individual contains the following knowledge: alert description, alert recommendation, alert source, alert date (last updated) and alert level (not recommended, contraindicated or not allowed prescription). We have not considered the lowest level of alert (to take into account) for reducing alert fatigue.

#### Local pharmacy ontology

**Fig. 4:**
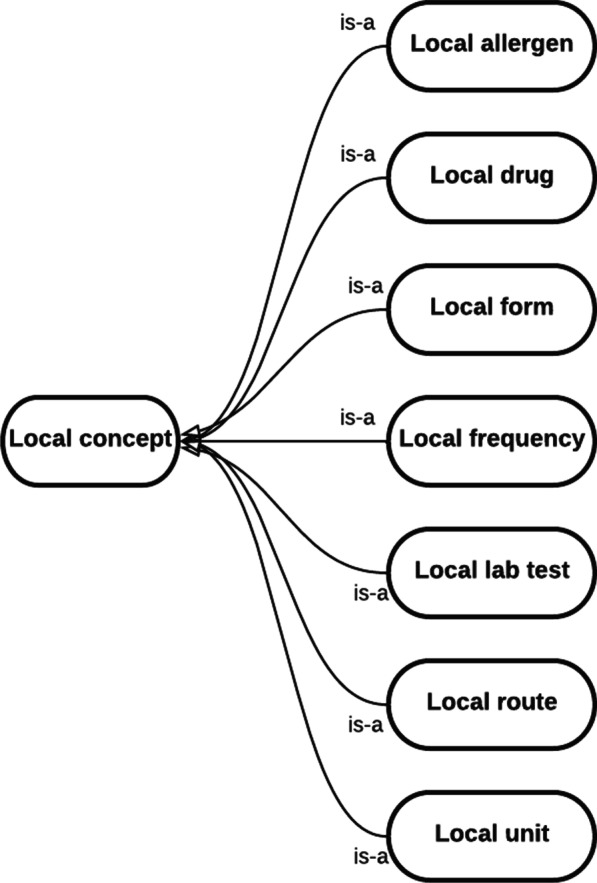
Diagram showing relationships between classes in local pharmacy ontology

Local pharmacy ontology was designed to represent local concepts from EHR and CPOE including local allergens, local drugs, local pharmaceutical forms, local frequencies, local lab tests, local administration routes and local units. Each local concept is mapped to the corresponding OntoPharma concept. Figure 4 provides a diagram showing the class interactions.

### Knowledge not represented using ontologies

Concerning maximum dose class, we did not represent maximum dose in the following situations: Maximum dose depends on the indication (acetylsalicylic acid as antiplatelet or analgesic). Maximum dose of an ingredient differs when administered alone or in combination (oral amoxicillin (6000 mg every 24 h); oral amoxicillin/clavulanic acid (2625 mg every 24 h). Maximum dose depends on pharmaceutical form with the same administration route (Hydroxycarbamide tablet (4200 mg every 24 h); Hydroxycarbamide capsule (9600 mg every 24 h).

Concerning renal adjustment class, we did not represent dosage for patients on dialysis or when the dosage depended on the indication. In addition, if the dosage recommended by clearing interval included several frequencies, we simplified to one (Dosage recommended: 500–2000 mg/8–12 h; Dosage represented: 1000–6000 mg/24 h).

### Ontology-driven alert module development

Once the patient-specific clinical data are sent from the CPOE/EHR to ontologies, local concepts are matched to their equivalent OntoPharma concepts. Depending on the type of alert, we defined the following decision rules.

To check that drugs do not exceed the recommended maximum dosage, total daily dose is calculated considering the dose, dose unit and frequency. We have defined conversion factors just in case the drug dose unit prescribed is different from the unit dose defined in the ontologies. If a maximum dose for an ingredient does not depend on the route of administration, all the doses prescribed for a same ingredient are added (parenteral acetaminophen 1000 mg/8 h and oral acetaminophen 500 mg/8 h is considered 3500 mg/24 h).

Drug–drug interactions checking considers the ATC codes of the drugs prescribed. Interaction is only considered if drugs overlap temporarily.

To evaluate prescription appropriateness in patients with renal failure, total daily dose is calculated in the same way as the maximum dosage checking. If a patient has several glomerular filtration rates values, we considered the most recent value.

In none of the previous cases medications prescribed “as needed” are considered.

Drug allergy checking considers if prescribed drugs are listed in the patient`s allergy history. Patient can be allergic to an entire drug class or to a specific drug. In the first instance, it is checked that none of the drugs included in the class are prescribed. Dose or administration route are not considered.

Regarding the interface, alerts are shown in different colours (red, orange, yellow) according to their clinical relevance. The advisory text contains the generic drug name and a short description of the possible concern. We defined such as soft-stop alerts those related with overdosing, drug-drug interactions, or dose adjustment for renal insufficiency. In these cases, the clinician can decide whether to ignore or to accept the alert. In case of acceptance and if there are more than two medications implied (Drug-drug interaction) clinician is asked about which medication wants to modify. To avoid alert fatigue, if an alert is ignored once, it will not display again.

The only hard-stop alerts defined was those related with an allergy. In these cases, the clinician cannot ignore the alert.

The interface which displays the alerts also includes a link to a user’s guide and an activity register which serves as traceability system.

The results show that the response time to generate decision support is of the order of milliseconds with the minimum impact on the workflow of the users.

### OntoPharma implementation

1046 patients were included. The median age was 69 (interquartile range (IQR) 52–81) years, and the majority were male (55.2%). The median length of hospital stay was 7 (IQR 4–13) days. Patients had a median number of 9 (IQR 6–12) medications.

574 (54.8%) patients received at least on alert. OntoPharma generated 823 alerts (mean of 1.4 ± 0.8 alerts per patient). 53 (6.4%) alerts were considered of low relevance, 761 (92.5%) as moderate and 9 (1.1%) as serious. 401 (48.7%) alerts were accepted.

Details of the type of alert and the acceptance rate are included in Table 2. The most frequently occurring alert was due to overdosing (47.1%), followed by inappropriate dosing in patients with kidney disease (30.3%), drug-drug interactions (21.5%), and allergy (1.1%).

**Table 2:** Description of the type of alerts generated by OntoPharma and the acceptance rate

Type of alert	Frequency n (%)	Acceptance rate n (%)
Maximum dosage alert	388 (47.1)	181 (46.6)
Drug-drug interaction checker	177 (21.5)	80 (45.2)
Renal failure adjustment	249 (30.3)	131 (52.6)
Drug allergy checker	9 (1.1)	9 (100.0)

## Discussion

This paper describes the design, development and implementation of OntoPharma, a CDSS for reducing medication prescribing errors based on ontologies.

The main innovation in this paper is the development of a knowledge based CDSS using ontologies instead of relational databases, which are the predominant choice in current commercial applications. Both data models consist of set of type definitions expressed in a formal notation. However, ontologies are focused on meaning and databases on data. Because ontologies add semantics to the models, they are more flexible and efficient to deal with changing and maintenance requirements than a database scheme [31, 32]. For example, interactions between drugs causing torsade de pointe could be implemented via a class “DrugCausingTorsadeDePointe”. In that case, when a new such drug is commercialized, one just have to add it to the class, while in a traditional database that lists pairs of interacting drugs, one have to add one pair for each already existing drug causing torsade de pointe, with the risk of forgetting one.

Ease in evolving ontologies is particularly important in the field of medicine considering that medical knowledge grows every day and CDSS have to reflect the current state of the underlying evidence to be effective [18]. It is also important to notice that explicitly declared knowledge can be used to argue proposed actions increasing user confidence in the CDSS.

In addition, semantic approach and the use of OWL enable a convenient infrastructure for reusing. By contrast, databases are designed mainly to meet the requirements of a particular application. This makes it hard to reuse a database when requirements change, resulting in higher maintenance costs [31, 32]. Despite ontologies can readily be reused, OntoPharma was developed from scratch because none of the existing ontologies met our needs.

Compared to commercial CDSS, most may contain a greater amount of drug data than OntoPharma. However, a high proportion of them are considered as basic, drug-oriented CDSS. Basic CDSS increase the risk of overriding an alert because of low specificity. The challenge to develop advanced CDSS which combines medication orders with patient characteristics, lies in ensuring interoperability [12]. To provide semantic interoperability we have integrated the use of ontologies and the use of a standardized controlled vocabulary such as SNOMED CT to encode medicinal products (VTM, VMP and VMPP) [33]. If an application uses a terminology different from the terminology used in OntoPharma, transformation mappings can be established using the ontology Local Pharmacy. Using ontologies, OntoPharma integrates structured clinical data with clinical knowledge, making a more refined and dynamic classification of patients in a mechanistic way [23].

Because of these features previously discussed, in the last decade there is an increasing interest in the use of ontologies based CDSS [24]. Some of them are addressed to medication management. However, they are focused on a specific subspecialty such as the management of chronic disease [34], cancer [35], antibiotic prescriptions [36], or diabetes [37], among others. In any case, it is not easy to make comparisons between ontologies. Although there are some guides about how to create ontologies, there is no one correct way to model a domain [38, 39]. The best solution depends on the final application and the extensions anticipated. For this reason, specialists in Medical Informatics have guided our ontologies design choices considering the clinician`s understanding and view of the medication domain. In addition, end users participation all along the design and development of OntoPharma ensures not interfere with their workflow and gain user acceptance [40, 41].

We have defined up to three ontologies. We have not combined everything into a single ontology for security and maintenance reasons. In this way, when we update A-Box we avoid making inadvertent mistakes in T-Box.

Ontologies enable operate on a higher level of abstraction so medication knowledge is represented in a more intuitive, extensible and maintainable manner in comparison with the initial dataset. For example, maximum daily dose source dataset contains various entries identified by the National Drug Code. In many cases, National Drug Codes are clinically equivalent pharmaceutical products with the same strength, dose form and the same routes of administration. We have defined maximum daily dose considering the ingredient and the route of administration. Thus, we have represented the same information using 562 individuals instead of 1013 entries. Similarly, we have defined drug-drug interactions considering ATC instead of National Drug Codes, so we have needed 2242 individuals instead of 3229 entries. We have also implemented the class "drug_route" which comprises all possible routes of administration. If, for example, the maximum dose of a drug is the same for all routes of administration, we only need one entry. In contrast, in traditional databases, an entry is needed for each route.To describe medication with a high level of detail, Drugs considers since the ingredient until the commercially produced packaged product. More than fifty properties defined at Drugs have been needed to represent all the technical data of medicinal products.

By contrast, we have failed to represent all the knowledge from the datasets containing appropriateness drug criteria. There is a wide variability among dosage recommendations in patients with renal failure, so we had to simplify the information. Any modification from the initial dataset was previously agreed with the advisory board. We have also not represented dosage for patients on dialysis or when the dosage depended on the indication because at the present time, dialysis modality or health problems are not structure fields in our EHR system. The wide adoption of electronic health record systems has led to the creation of large amounts of healthcare data. However, much of the patient data, especially reasons for clinicians’ decisions, are in unstructured text format. Thus, there is an urgent need for an automated way that converts free text data into structured fields computationally [42, 43].

Another limitation of our study is to keep up with evidence. To date, we incorporate information from different sources including regulatory agencies and local practice-based evidence. However, evidence review is an extremely demanding and time-consuming process and often not easy to come by. Automatic update from resources for evidence-based medicine is an active area of inquiry [44]. We consider that is a critical priority that has not been resolve.

Similarly, manual mapping local terms to standard vocabularies is challenging. Specific domain knowledge and knowledge of the target vocabulary standards is required to ensure high clinical quality mappings. In addition, maintain the manual mappings is a resource-intensive and ongoing process. Although there are tools for auto-mapping, the clinical domain is complex and expert human review is still needed.

Because of clinician’s limited time and attention, another challenge of OntoPharma is to display alerts based on user purpose and preferences [45, 46]. We have already started working on customisation according to user needs.

In terms of evaluation, the proposed system has not been compared directly to classical database-based systems. Testing retroactively the patient data with a commercial drug database could have provided interesting comparative data. In practice, we know 49–96% of alerts generated by CDSS based on relational databases are overridden [14].Findings from other studies provide evidence of the potential usefulness of ontologies for improving alert generation. However, our acceptance rate was lower than expected (49%). We have showed that ontologies can replace database for drug knowledge, but not really that they can do better. It may be partly explained by the limitations mentioned above. Furthermore, we are aware that the alerts generated by OntoPharma are nowadays commonly available. In fact, we have modelled drug knowledge using information contained in databases, so we have not taken advantage of all the potential of ontologies. For instance, interactions between more than two drugs do exist, but they are almost never considered in knowledge sources. In addition, OntoPharma has been implemented in a tertiary referral hospital. We have not considered the care delivery setting for which the CDSS is applicable, so alerts for stable outpatients were overridden. We are currently placing a high priority on specifying the clinical context and constructing models of scalable specificity. We also have to consider that evaluations from lots of studies using ontologies occurred in a controlled environment, thus usefulness might vary in a clinical setting [47, 48]. Another aspect to consider is the usability of OntoPharma. We have not received any complaints from users. We believe that active involvement of users and iterative design solutions during the development stage ensure satisfaction of the end-users. However, in future, it would be interesting to conduct formal usability testing.

In any case, it is necessary to investigate not only the factors influencing alert acceptance but the impact of OntoPharma on health outcomes too.

Medication safety is a high complexity problem that requires a progressive approach. Despite the results are close to those obtained in the existent database-based systems, ontology-based approach is more efficient to deal with complexity than a traditional system. For this reason, the work described in this paper is only a starting point for developing more complex use cases focus on specific populations [49]. Currently we are representing complex drug knowledge absent in usual commercial databases for supporting older patients with chronic conditions and for neonatal population. In future, based on structured patient data, knowledge representation could also offer advice to aid diagnostic and therapeutic decision-making instead of detect medication errors once occurred.

Considering that OntoPharma is restricted to the medication prescription process, developing a more complex CDSS that applies across the entire treatment process is also desirable for future work.

On the other hand, the use of OWL lead to other benefits adding reasoning capabilities which could provide new knowledge, a promising avenue for future research [23].

We recognize that the best ontology based CDSS will not probably replace clinicians [50]. Clinical reasoning is a context-dependent complex process that requires taking into account many factors to guide practice actions. Despite the limitations, we consider that developing ontologies to support the medication domain knowledge is a great first step for reducing medication errors.

## Conclusions

Medication prescribing errors are a significant global health problem with important impact in patient safety. OntoPharma, an ontology based CDSS, solves some of the challenges of CDSS based on relational databases and provides more flexibility, scalability and sustainability. Our approach has been designed and developed by a multidisciplinary team to reflect the expert’s view. OntoPharma is implemented in clinical practice and generates alerts when a prescribing medication error is identified according to patient data. Using ontologies as the knowledge base of a CDSS provides a good opportunity to reduce medication errors.

## Supplementary Information

**Additional file 1.** Medication knowledge concepts represented in OntoPharma. Full list of the medication knowledge concepts and their definitions represented in OntoPharma.

**Additional file 2.** Properties and their facets represented in OntoPharma. Full list of the properties and their facets represented in OntoPharma.

## Data Availability

The datasets generated and/or analysed during the current study are not publicly available due to their containing information that could compromise the privacy of research participants but are available from the corresponding author on reasonable request.

## Abbreviations

AEMPS: Spanish agency of medicines and medical devices
AMP: Actual medicinal product
AMPP: Actual medicinal product pack
API: Actual product ingredient
AQuAS: Agency for Health Quality and Assessment of Catalonia
ATC: Anatomical therapeutic chemical code
CDSS: Clinical decision support system
CPOE: Computerised physician order entry
DSS: Decision support system
EHR: Electronic health record
IQR: Interquartile range
OWL: Web ontology language
VMP: Virtual medicinal product
VMPP: Virtual medicinal product pack
VTM: Virtual therapeutic moiety
