# Supplementary material for: OntoPharma: ontology based clinical decision support system to reduce medication prescribing errors
Source: BMC Med Inform Decis Mak. 2022 Sep 10;22:238. doi: 10.1186/s12911-022-01979-3 (PMC9463735; doi:10.1186/s12911-022-01979-3)
Supplement: Supplementary file 1 — Additional file 1. Medication knowledge concepts represented in OntoPharma. Full list of the medication knowledge concepts and their definitions represented in OntoPharma. [file 12911_2022_1979_MOESM1_ESM.docx]

| **Additional file 1: Medication knowledge concepts represented in OntoPharma** | |
| --- | --- |
| **Drugs ontology** | |
| **Concept** | **Concept definition** |
| Active ingredient | Any substance or combination of substances used in a pharmaceutical product, intended to furnish pharmacological activity or to otherwise have direct effect in the diagnosis, cure, mitigation, treatment or prevention of disease. |
| AI base | Active ingredient as the core or base molecule |
| AI derivative | The different salts, esters, ethers, isomers, mixtures of isomers, complexes or derivatives of an active ingredient |
| AMP | An actual medicinal product is a medicinal product that has been made available by a supplier. It is the medicinal product that is taken by a patient |
| AMPP | An actual medicinal product pack is the commercially produced packaged product which is supplied for direct patient use. The concept contains information on the pack size, the inner packaging, price and reimbursement information, and other administrative information linked to the concept |
| API | An actual product ingredient is an ingredient used in an actual medicinal product |
| ATC | Drugs grouped by the Anatomical Therapeutic Chemical code |
| Chemical group | Drugs grouped by their pharmacologic class |
| Composition unit | Minimum unit in which the dose is administered |
| Drug | A medicine or other substance which has a physiological effect when ingested or otherwise introduced into the body |
| Drug dose form | The physical form of a dose of a drug |
| Drug route | Route of administration for drugs |
| Excipient | An inactive substance that serves as the vehicle or medium for a drug |
| Matter | [Material](https://dictionary.cambridge.org/es-LA/dictionary/english/material) with [particular](https://dictionary.cambridge.org/es-LA/dictionary/english/particular) [physical](https://dictionary.cambridge.org/es-LA/dictionary/english/physical) [characteristics](https://dictionary.cambridge.org/es-LA/dictionary/english/characteristic) |
| Medicinal product | A medicine or other substance which has a physiological effect when ingested or otherwise introduced into the body |
| Pack | The packaging of [medicinal products](https://www.ema.europa.eu/en/glossary/medicinal-product) |
| Packaged medicinal product | Abstract concept representing one or more quantitatively equivalent actual medicinal product packs |
| Pharmacological group | Drugs grouped by the Anatomical Therapeutic Chemical code (ATC). ATC is a unique code assigned to a medicine by the World Health Organization according to the organ or system it works on and how it works. In the ATC classification system, the active substances are classified in a hierarchy with five different levels.  The system has fourteen main anatomical/pharmacological groups or 1st levels.  Each ATC main group is divided into 2nd levels which could be either pharmacological or therapeutic groups.  The 3rd and 4th levels are chemical, pharmacological or therapeutic subgroups and the 5th level is the chemical substance |
| Presentation unit | Unit of measurement containing the dose of active ingredient |
| Product ingredient | Any substance or combination of substances used in a pharmaceutical product, intended to furnish pharmacological activity or to otherwise have direct effect in the diagnosis, cure, mitigation, treatment or prevention of disease. |
| State | Marketing authorization status of the medicinal product |
| Strength unit | Unit of measurement for the active ingredient quantity |
| Units | Units of measure |
| Unit equivalence | Conversions between units |
| VMP | A virtual therapeutic moiety is an abstract representation of an active medicinal ingredient or substance devoid of strength and form, which when formulated as a medicinal product, is intended for use in preventing or treating diseases in patients |
| VMPP | A virtual medicinal product is a representation of a VTM associated with strength information and a route of administration. It represents a collection of clinically equivalent pharmaceutical products with the same strength, dose form and the same routes of administration. |
| VPI | A virtual product ingredient is an ingredient used in a virtual medicinal product |
| VTM | A virtual therapeutic moiety is an abstract representation of an active medicinal ingredient or substance devoid of strength and form, which when formulated as a medicinal product, is intended for use in preventing or treating diseases in patients. |
| **DSS ontology** | |
| **Concept** | **Concept definition** |
| Alert | Types of alerts |
| Alert description | Text describing the causes of medication errors |
| Alert level | Alert levels according to the clinical relevance |
| Alert recommendation | Text describing the recommendation to avoid a medication error |
| Appropriateness criteria | [Prescribing](https://www.lawinsider.com/dictionary/therapeutic-appropriateness) criteria based on scientific evidence |
| Appropriateness lab test | Drug appropriateness criteria based on a laboratory test |
| Dose adjustment | Drug appropriateness criteria based on dose adjustment guided by a laboratory test |
| Dose appropriateness | Drug appropriateness criteria based on dose adjustment |
| Drug appropriateness | [Drug prescribing](https://www.lawinsider.com/dictionary/therapeutic-appropriateness) criteria based on scientific evidence |
| Drug interaction | Interactions of drugs with other drugs |
| Lab test | Types of laboratory test |
| Maximum dose | Drug appropriateness criteria based on maximum dose adjustment |
| Maximum dose adult | Maximum dosage in adults |
| Multilingual text | Text shown in the alerts |
| Renal adjustment | Drug dosing based on kidney function |
| **Local pharmacy ontology** | |
| **Concept** | **Concept definition** |
| Local allergens | Allergens used in the local environment |
| Local concepts | Concepts used in the local environment |
| Local drugs | Drugs used in the local environment |
| Local forms | Types of dosage forms used in the local environment |
| Local frequencies | Frequencies (how often the medication is to be administered as events per unit of time) used in the local environment |
| Local lab tests | Laboratory tests used in the local environment |
| Local routes | Route of administration for drugs used in the local environment |
| Local units | Units of measure used in the local environment |
