# Supplementary material for: OntoPharma: ontology based clinical decision support system to reduce medication prescribing errors
Source: BMC Med Inform Decis Mak. 2022 Sep 10;22:238. doi: 10.1186/s12911-022-01979-3 (PMC9463735; doi:10.1186/s12911-022-01979-3)
Supplement: Supplementary file 2 — Additional file 2. Properties and their facets represented in OntoPharma. Full list of the properties and their facets represented in OntoPharma. [file 12911_2022_1979_MOESM2_ESM.docx]

| **Additional file 2: Properties and their facets represented in OntoPharma** | | | |
| --- | --- | --- | --- |
| **Drugs** | | | |
| **Domain** | **Property** | **Range** | **Object (O) or Datatype (D)** |
| AMP | API | Product_Ingredient | O |
|  | excipient | Excipient | O |
|  | market_lab | - | O |
|  | owner_lab | - | O |
|  | reg_num | int | D |
| AMPP | affects_driving | Boolean | D |
|  | Amp | AMP | O |
|  | ampp_authorization_date | Date | D |
|  | ampp_state | State | O |
|  | ampp_state_date | Date | D |
|  | biosimilar | Boolean | D |
|  | black_triangle | Boolean | D |
|  | clinic_packaging | Boolean | D |
|  | discharge_prescription | Boolean | D |
|  | generic_drug | Boolean | D |
|  | hospital_diagnose | Boolean | D |
|  | hospital_use | Boolean | D |
|  | long_duration | Boolean | D |
|  | marketed | Boolean | D |
|  | marketed_date | Date | D |
|  | narcotic | Boolean | D |
|  | national_code | int | D |
|  | orphan | Boolean | D |
|  | plant_based | Boolean | D |
|  | prescription | Boolean | D |
|  | psychotropic | Boolean | D |
|  | replaceable | Boolean | D |
|  | special_control | Boolean | D |
| Pharmacological_group | group_code | String | D |
| Product_Ingredient | active_ingredient | Active_ingredient | O |
|  | comp_unit | Unit | O |
|  | presentation_unit | Unit | O |
|  | strength_unit | Unit | O |
|  | strength_comp | Float | D |
|  | strength_presentation | Float | D |
| VMP | dose_form | Drug_dose_form_type | O |
|  | drug_route | Drug_route_type | O |
|  | main_route | Drug_route_type | O |
|  | multi-dose | Boolean | D |
|  | multi-ingredient | Boolean | D |
|  | Vpi | Product_Ingredient | O |
| VMPP | content_unit | Unit | O |
|  | content_quantity | Float | D |
|  | pack_unit | Pack_type | O |
|  | quantity_dispen_unit | Float | D |
|  | unit_quantity_dispen_unit | Unit | O |
|  | Vmp | VMP | O |
| VTM | has_active_ingredient | Active_ingredient | O |
| **DSS** | | | |
| **Domain** | **Property** | **Range** | **Object or Datatype** |
| Alert | alert_date | String | D |
|  | alert_description | Alert_description | O |
|  | related_information | anyURI | D |
|  | alert_level | Alert_level | O |
|  | alert_recommendation | Alert_recommendation | O |
|  | alert_source | String | D |
| Alert_level | level | int | D |
| Appropriateness_criteria | alert | Alert | O |
| Appropriateness_lab_test | lab_test | Lab_test_type | O |
|  | Lab_test_unit | dmm:Unit | O |
|  | high_value | Float | D |
|  | low_value | Float | D |
| Dose_adjustment | adjusted_base_unit | dmm:Unit | O |
|  | adjusted_dose_unit | dmm:Unit | O |
|  | adjusted_loading_base_unit | dmm:Unit | O |
|  | adjusted_loading_dose | Float | D |
|  | adjusted_loading_unit | dmm:Unit | O |
|  | max_adjusted_dose | Float | D |
|  | min_adjusted_dose | Float | D |
| Dose_appropriateness | base_unit | dmm:Unit | O |
|  | dose_unit | dmm:Unit | O |
|  | drug | dmm:Drug | O |
|  | max_age | Float | D |
|  | min_age | Float | D |
| Drug_appropriateness | ingredient | dmm:Ingredient | O |
|  | route | dmm:Drug_route_type | O |
| Drug_Interaction | drug_1 | dmm:Pharmacological_group | O |
|  | drug_2 | dmm:Pharmacological_group | O |
| Maximum_dose | max_dose | Float | D |
| **Local_Pharmacy** | | | |
| **Domain** | **Property** | **Range** | **Object or Datatype** |
| Local_concept | local_code | String | D |
|  | ontopharma_concept | dmm:Ontopharma_concept | O |
| Local_frequency | daily_frequency | Float | D |
| Local_lab test type | validity_period | Float | D |
|  | validity_period_unit | dmm:Unit | O |
